# Comparative cross-sectional study of empathy among first year and final year medical students in Jimma University, Ethiopia: Steady state of the heart and opening of the eyes

**DOI:** 10.1186/1472-6920-12-34

**Published:** 2012-05-24

**Authors:** Sandra Dehning, Eshetu Girma, Sarah Gasperi, Sebastian Meyer, Markos Tesfaye, Matthias Siebeck

**Affiliations:** 1 Department of Psychiatry and Psychotherapy, Ludwig-Maximilians-University, Munich, Germany; 2 Department of Health Education and Behavioral Sciences, Jimma University, Jimma, Ethiopia; 3 Department of Psychiatry, Jimma University, Jimma, Ethiopia; 4 Department of Surgery, Ludwig-Maximilians-University, Munich, Germany

**Keywords:** Cognitive empathy, Emotional empathy, Medical education, Jimma University

## Abstract

**Background:**

There is general consent that empathy is crucial for the physician-patient relationship and thus an important issue in medical education. This comparative study was designed to examine the differences in empathy between first year and final year medical students in Jimma University, Ethiopia.

**Methods:**

A comparative cross-sectional study among 131 first year and 106 final year medical students was conducted in Jimma University, Ethiopia on academic year 2010/11. The study subjects were selected using simple random sampling technique from the list of the students. Study participation was voluntary. The Balanced Emotional Empathy Scale (BEES) was used for the detection of “heart-reading”, i.e. emotional empathy and the Reading the Mind in the Eyes test (RME-R test) to evaluate “mind-reading”, i.e. cognitive empathy. We performed *t*-test to compare the mean difference in empathy and RME-R scores between the two groups of students. A linear regression was computed to identify potential factors influencing the BEES and RME-R.

**Results:**

Out of the total 237 students, 207 (87.3%) were males. The mean age of first year and final year students was 19.3 ± 1.1 and 24.0 ± 1.4 years respectively. First year students have scored 40.6 ± 23.8 while final year students scored 41.5 ± 20.8 mean in the BEES measuring emotional empathy score. However, this difference was not statistically significant (t = −0.30, df = 231, P-value >0.05). Final year students had significantly higher mean cognitive empathy score (17.8 ± 4.5) than first year students (14.4 ± 4.8) [β = 2.7, 95%CI (1.20, 4.13)]. Males had scored lower cognitive [β = −2.5, 95%CI (−4.37, −0.66)] and emotional empathy [β = −12.0, 95%CI (−21.66, −5.46)].

**Conclusions:**

Low emotional (BEES) and cognitive empathy sores were found in first year and final year students of Jimma University could have implications on the medical education curricula. Medical education targeted at enhancing emotional empathy and increasing cognitive empathy is required by segmenting with gender for effective physician-patient interaction. The influence of empathy on clinical competence should be studied using more rigorous design.

## Background

It was in 1977 in Australia when empathy in medical students was measured for the first time [1]. Since then, plenty of investigations into empathy at medical schools all over the world followed [2-8], so far with no consideration of the continent of Africa. Empathy has been described as a concept involving cognitive as well as emotional domains [9]. The cognitive domain of empathy involves the ability to understand another person’s inner experiences and feelings and a capability to view the outside world from the other person’s perspective [10]. Such a cognitive component is also amenable to training and, thus, medical schools can play a positive role in the development of students’ understanding about empathy [4].

The emotional domain involves the capacity to enter into or join the experiences and feelings of another person [10,11]. The emotional relationships that elicit emotional response are conceptually more relevant to sympathy than to empathy [12]. Because sympathy, if excessive, could interfere with objectivity in diagnosis and treatment [11,13], “compassionate detachment” has been used to describe the physician’s empathetic concern for the patient while keeping sympathy at a reasonable distance to maintain an emotional balance [13,14]. Hence, an “emotional distance” would be desirable to avoid bursts of emotions that might interfere with clinical neutrality and personal durability [15].

Main findings of studies recorded a decline in empathy during medical school proceedings [3-5,16-22], a higher empathy level in females compared to males [4,5,7,16,19,21-26], and a relation between the students` choice of the future medical specialization and their empathy level scores [4,22,26,27]. The disturbing possibility is that medical education might be injuring instead of nurturing empathy [28]

There is general consent that empathy is crucial for the physician-patient relationship and thus an important issue in medical education. Empathy represents the “touch” in modern medicine, at present ill-reputed as “high tech, low touch” [29]. As the theoretical constructs of empathy are complex, physicians’ appropriate empathy is still under discussion. Jodi Halpern suggested an answer to the question “What is clinical empathy?” considering the attention to the patient as the focus of the physicians’ task, not seeing any necessity of experiencing vicariously their patients’ emotions [30]. Depending upon developmental, experiential, social, educational, and other endogenous and exogenous factors, one group may possess more or less empathy than another group [31].

So far, there is no study of empathy among medical students in Ethiopia; taking into account possible cultural influences on medical students’ empathy or other empathy-influencing factors like socio-demographic and personal characteristics. In addition to experiences in clinical practice, since medical students in the University take courses on behavioral sciences, we expect final year students to have less emotional empathy and higher cognitive empathy than first year students. Hence, this study was intended to assess whether empathy increases with medical training and identify the socio-demographic background of medical students influencing their empathy level

## Methods

Comparative cross-sectional study among 131 first year and 106 final year (fifth year) medical students was conducted in Jimma University, Ethiopia on academic year 2010/11. Jimma University is found in Jimma city located 350 km southwest of Addis Ababa, Ethiopia. There were about 1000 medical students in total, the number of first-year students being around 210 and final year students around 150. Assuming small to medium effect sizes (Cohen’s d = 0.4) and a power of 80%, a sample size of 100 students per group was envisaged. In the end, 131 first year and 106 final year medical students were included in the study. The study subjects were selected using simple random sampling technique from the list of the students.

Two different self administered survey instruments were used to measure the students’ empathy. The Balanced Emotional Empathy Scale (BEES) was used for the measurement of “heart-reading”, i.e. emotional empathy [32]. The BEES is an instrument consisting of 15 positively and 15 negatively worded items that measure emotional responses to fictive situations and particular life events (examples of the items include: “Unhappy movie endings haunt me for hours afterward, I cannot feel much sorrow for those who are responsible for their own misery”). The coefficient alpha internal consistency of the BEES is 0.87. The questions attempt to probe the extent to which the respondent is able to feel the other’s suffering or take pleasure in their happiness. Study subjects report the degree of their agreement or disagreement for each of the 30 items using a 9-point Likert-scale. A higher total score represents a higher level of emotional empathy. The stated norm provided in the Manual for the BEES is 45 ± 24 [32].

As gaze perception plays a crucial role in the ability to reason about others’ intentions and feelings [33], the Reading the Mind in the Eyes test (RME-R test) was used to evaluate “mind-reading”, i.e. cognitive empathy. The RME-R test consists of 36 photographs depicting just the eye regions. A rectangular area of approximately 5 × 2 in. delineated the eye region, encompassing the entire width of the face from midway up the nose to right above the brow. Four mental states accompanying each stimulus (one target word and three foils) were presented at each corner of the high resolution photograph. To reduce linguistic difficulties, the test had appended a detailed glossary where all adjectives were explained using synonyms and example sentences. A typical mean score is in the range 22–30. A mean score over 30 indicates a very accurate at decoding a person’s facial expressions around the eyes. A score under 22 indicates a very low score of RME-R test [34].

Socio-demographic characteristics included questions about gender, age, year of education, ethnicity, country of birth, migration background, the people with whom the students grew up (mother/father/both/other), number of siblings, position (birth order) within siblings (eldest/sandwich/youngest), major life events during childhood (divorce/illness/death of parents), place of residence (at home with relatives/moved out), socio-economic status of parents, religion, active membership in a religious community, number of close relationships, involvement in online social networks like facebook, daily internet use, interest in a medical specialization (specialization with continuity of patient care such as internal medicine, psychiatry and pediatrics versus specialization with less interpersonal contact such as surgery, radiology and pathology) and a question about students’ personal experience with psychiatric or psychotherapeutic treatment.

To deal with missing values in the self-rating scale BEES, imputation by the individual mean of the observed items was applied separately for the positively and the negatively worded items, in case the respective number of missing values did not exceed 5 (i.e. 33%). Otherwise the questionnaire was treated as insufficient and was excluded from the analyses. In the RME-R test, missing answers were treated as “the participant did not recognize the emotion”. However, if more than the half of the RME-R questionnaire was empty, this was interpreted as insufficient motivation to complete the test, which was therefore excluded from the analyses. Apart from descriptive statistics, *t*-test for empathy and RME-R scores were performed to check for mean difference between the two groups of students. Pearson correlations between the BEES and the RME-R were also calculated. A linear regression was computed between the empathy scores and socio-demographic and other background characteristics using the enter method. A significance level of 5% was used. All analyses were performed using SPSS version 16.

Ethical approval for the study was granted by the Ethics Review Board of Jimma University, college of public health and Medical Sciences. After a brief explanation of the study, a written consent was obtained from each participant.

## Results

### Characteristics of the study participants

Out of the total 237 students, 207 (87.3%) were males. The mean ages of first year and final year students were 19.3 ± 1.1 and 24.0 ± 1.4 years respectively. The combined mean age was 21.4 ± 2.7 years. Majority of them 119(50.2%) were Oromo in ethnicity and Christian religion followers, 177(74.7%). Most of the students195 (82.3%) were living with both of their parents. More than half 118(52.4%) of the students were neither the youngest nor the eldest child in their family (i.e. are sandwich) and were living with their family or relatives; 131(58.2%). A large proportion of the students 156(67.2%) perceived themselves as active members of their religion. Only 77(32.9%) of them have ever faced major life events (divorce/illness/death of parents) during their childhood and 7(3.0%) had a history of migration. Majority 141(60.3) of the students were currently using online social media like facebook as a social media. Small proportion 27(12.3%) have never used internet. Most of them 147(63.4%) were interested in specialization with continuity of patient care (i.e. specializations which have more interactions with patients). Sixteen (6.8%) of the students had a history of psychiatric treatment (Table 1).

**Table 1:** Socio-demographic and background characteristics of first year and final year medical students of Jimma University; Ethiopia, 2011

**Variable**		**Year No(%)**		**P-value**
		**First year (n_1_ = 131)**	**Final year (n_2_ = 106)**	
**Sex**	Male	115(87.8)	92(86.8)	0.91
	Female	16(12.2)	14(13.2)	
**Ethnicity**	Oromo	52(39.7)	67(63.2)	0.01
	Amhara	46(35.1)	28(26.4)	
	Others	33(25.4)	11(10.4)	
**Grow up with**	Mother	8(6.1)	11(10.4)	0.01
	Father	3(2.3)	4(3.8)	
	Both	108(82.4)	87(82.1)	
	Other	12(9.2)	4(3.8)	
**Position in the family**	Eldest	32(26.4)	33(31.7)	0.18
	sandwich	61(50.8)	57(54.8)	
	youngest	28(23.3)	14(13.5)	
**Place of living**	At home with family or relatives	71(57.7)	60(58.8)	0.68
	Not at home	52(42.3)	42(41.2)	
**Working status of mother**	working	49(39.8)	52(53.1)	0.30
	housewife	66(53.7)	40(40.8)	
	out of work	8(6.5)	6(6.1)	
**Working status of father**	working	101(82.8)	84(87.5)	0.01
	houseman	6(4.9)	8(8.3)	
	out of work	15(12.3)	4(4.2)	
**Religion**	Christian	101(77.1)	76(71.7)	0.32
	Muslim	28(21.4)	29(27.4)	
	Other	2(1.5)	1(0.9)	
**Religion active member**	yes	95(73.6)	61(59.2)	0.06
	No	34(26.4)	42(40.8)	
**Major life event**	yes	45(34.9)	32(30.5)	0.30
	no	84(65.1)	73(69.5)	
**Online social media like facebook**	yes	51(39.8)	90(84.9)	<0.001
	No	77(60.2)	16(15.1)	
**Migration history**	Yes	1(0.8)	6(5.7)	0.09
	No	129(99.2)	100(94.3)	
**Daily internet time**	less than 1 h	90(77.6)	74(71.2)	0.04
	more than 1 h	11(9.5)	18(17.3)	
	no internet	15(12.9)	12(11.5)	
**Interested in**	continuity of patient care	75(58.6)	72(69.2)	0.47
	less interpersonal contact	31(24.2)	18(17.3)	
	no idea	22(17.2)	14(13.5)	
**History of Psychiatric treatment**	yes	7(5.5)	9(8.5)	0.65
	No	121(94.5)	97(91.5)	

*For some of the variables the individual sum is not equal to the total sample size since there are missing values.

### Emotional empathy

**Figure 1:**
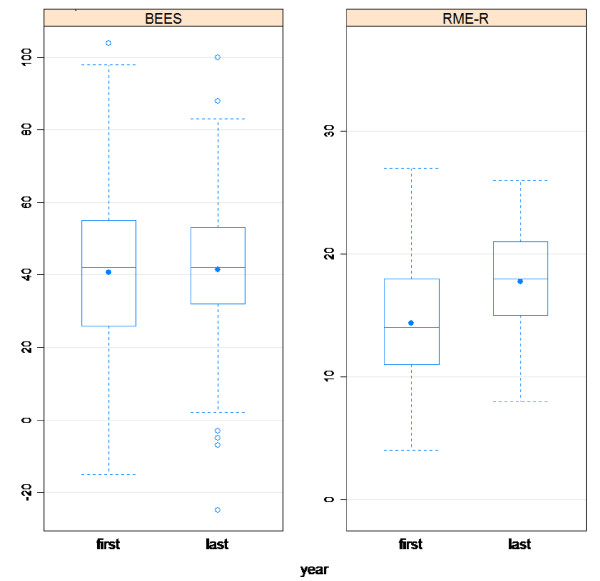
Box plot showing BEES and RME-R scores of first year and final year students of Jimma University; Ethiopia, 2011.

First year students had mean BEES score of 39.0 ± 22.3 for male and 51.8 ± 30.6 for female. Final year students had mean BEES score of 39.9 ± 20.0 for male and 51.5 ± 23.5 for female. Male students had statistically significant lower mean BEES score (t = −2.81, df = 233, p-value < 0.05). First year as well as final year students who were using social media like facebook has scored higher mean emotional empathy scores (45.2 ± 24.1 and 42.6 ± 21.3) respectively. Use of social media like facebook had significant statistical association (t = 2.20, df = 195, P-value <0.05) with mean emotional empathy score. Generally, first year students have scored 40.6 ± 23.8 while final year students have scored 41.5 ± 20.8 mean emotional empathy (Table 2 ). The median emotional empathy score was also almost similar for both groups of students (Figure 1). Also, there was no statistically significant difference between first year and final year medical students on the mean emotional empathy score (t = −0.30, df = 231, P-value >0.05).

**Table 2:** Mean and standard deviation score of BEES of first year and final year medical students of Jimma University; Ethiopia, 2011

**Variable**		**Mean BEES (SD)**		**P-value**
		**First year (n_1_ = 131)**	**Final year (n_2_ = 106)**	
**Sex**	Male	39.0(22.3)	39.9(20.0)	0.91
	Female	51.8(30.6)	51.5(23.5)	
**Ethnicity**	Oromo	38.4(23.2)	39.3(21.8)	0.01
	Amhara	38.9(25.3)	46.1(20.2)	
	Others	46.3(22.2)	43.0(14.1)	
**Grow up with**	Mother	37.3(17.6)	38.6(20.3)	0.72
	Father	37.3(2.5)	54.0(31.6)	
	Both	40.1(24.3)	41.4(20.6)	
	Other	47.6(25.3)	39.3(18.7)	
**Position in the family**	Eldest	48.2(18.8)	38.0(23.5)	0.24
	sandwich	41.3(24.0)	43.3(18.6)	
	youngest	32.2(27.8)	42.8(23.2)	
**Place of living**	At home with family or relatives	41.9(24.1)	43.2(19.1)	0.16
	Not at home	39.0(24.0)	37.5(22.6)	
**Working status of mother**	working	39.6(26.7)	42.3(20.9)	0.86
	housewife	41.4(21.5)	39.3(19.4)	
	out of work	37.8(26.4)	37.3(15.2)	
**Working status of father**	working	40.5(24.6)	40.1(20.8)	0.98
	houseman	29.5(17.6)	49.4(30.9)	
	out of work	41.1(18.8)	39.0(8.0)	
**Religion**	Christian	39.7(24.6)	40.8(20.0)	0.54
	Muslim	42.9(21.3)	43.2(23.3)	
	Other	53.5(9.2)	41.0(0.0)	
**Religion active member**	Yes	44.0(23.1)	40.2(20.1)	0.18
	No	32.7(23.5)	42.8(22.3)	
**Major life event**	Yes	44.8(22.4)	39.9(21.9)	0.32
	No	37.8(24.1)	42.0(20.5)	
**Online social media like facebook**	Yes	45.2(24.1)	42.6(21.3)	0.03
	No	37.2(22.8)	35.2(16.9)	
**Migration history**	Yes	98.0(0.0)	33.2(28.0)	0.87
	No	40.2(23.3)	42.0(20.3)	
**Daily internet time**	less than 1 h	39.7(24.1)	42.2(22.5)	0.85
	more than 1 h	36.0(27.8)	40.2(17.8)	
	no internet	39.6(19.5)	37.7(15.2)	
**Interested in**	Continuity of patient care	41.8(23.9)	43.0(20.4)	0.10
	less interpersonal contact	40.8(24.6)	43.9(20.0)	
	no idea	32.7(20.0)	35.1(21.1)	
**History of Psychiatric treatment**	Yes	26.3(22.9)	37.9(19.4)	0.12
	No	41.7(23.7)	41.8(21.0)	
**Total BEES mean (SD)**	40.6(23.8)	41.5(20.8)	41.1(22.4)	0.81

The regression analysis has indicated that first year male students had less emotional empathy [β = −13.7, 95%CI (−27.22, −0.19)] than first year female students. There was no significant statistical difference in emotional empathy score with the remaining socio-demographic characteristics of first year students. There was no significant statistical emotional empathy score difference with any of the socio-demographic characteristics among final year students. The overall regression analysis indicated that male compared with female [β = −12.0, 95%CI (−21.66, −5.46)] and those who have not decided about future specialization interest area compared with who decided [β = −11.12, 95%CI (−20.91, −1.34)] had less emotional empathy. Students who were using social media like facebook had significantly higher emotional empathy score than non-users [β = 11.8, 95%CI (4.05, 19.43)] (Table 3).

**Table 3:** Predictors of BEES score of first year and final year medical students of Jimma University; Ethiopia, 2011

**Variables**	**β**	**Std. Error P-value**	**95% Confidence Interval for β**	
			**Lower Bound**	**Upper Bound**
**Number of brothers and sisters**	−0.84	0.58	−1.99	0.31
**Religion** (Reference = Muslim)				
Christian	−3.62	3.91	−11.34	4.10
Other	5.38	13.83	−21.88	32.65
**Sex** (Reference = female)				
Male	−12.03	4.89	−21.66	−2.39
**Year of study** (Reference = first)				
Second year	5.72	7.63	−9.32	20.76
**Migration history (reference = yes)**				
No	6.05	9.05	−11.80	23.90
**Grown up with (Reference = Mother)**				
Father	0.35	2.14	0.16	0.87
Both	1.33	1.20	1.10	0.27
Other	−0.10	1.71	−0.06	0.95
**Position in the family** (Reference = Eldest)				
Sandwich	5.76	3.57	−1.29	12.81
Youngest	−3.76	4.65	−12.93	5.42
**Major life event** (reference = No)				
Yes	2.65	3.65	−4.55	9.85
**Mothers job** (reference = working)				
Housewife	2.23	3.37	−4.42	8.88
Out of work	5.03	7.56	−9.87	19.93
**Fathers job** (reference = working)				
Houseman	5.49	6.40	−7.14	18.11
Out of work	−4.12	9.55	−22.95	14.70
Religion active member **(reference = No)**				
Yes	0.620	3.31	−5.92	7.16
**Use of social media like facebook** (reference = No)				
Yes	11.75	3.90	4.05	19.43
**Daily internet use** (reference = no internet)				
Less than 1 h	−7.23	4.43	−15.97	1.51
more than 1 h	−10.81	6.15	−22.95	1.33
**Interested in (reference = less interpersonal contact)**				
in patient care	1.97	3.75	−5.43	9.36
No idea	−11.12	4.96	−20.91	−1.34
**History of psychiatric treatment** (reference = No)				
Yes	−5.82	5.89	−17.44	5.80
R^2^ = 0.18				

### Cognitive empathy

Male students have scored lower mean cognitive empathy score than females both in first year and final year (14.2 ± 4.7 for males Vs 16.3 ± 4.8 for females) and (17.2 ± 4.3 for males Vs 21.2 ± 4.0 for females) respectively. Sex and cognitive empathy had significant statistical association (t = −3.12, df = 37, P-value < 0.05). Age of the students had statistically significant positive correlation with cognitive empathy score (Pearson correlation (r) =0.26, P-value < 0.05). The number of brothers and sisters students have had statistically significant negative correlation with cognitive empathy score of the students (Pearson correlation (r) = −0.26, P-value < 0.05).

Both class of students living at home with family or relatives had higher mean cognitive empathy scores (14.9 ± 4.8 for first year and 18.6 ± 4.6 for final year) than those who lived alone. Whether students are living at home or not had significant statistical association (t = 2.25, df = 207, P-value < 0.05). Both first year and final year students who were using social media like facebook have scored higher mean cognitive empathy (15.8 ± 4.4 for first year and 17.8 ± 4.5 for final year students). Use of social media like facebook (t = 4.45, df = 177, P-value < 0.05) and mothers socioeconomic condition (t = 3.15, df = 199, P-value < 0.05) had significant statistical association. Final year students had (17.8 ± 4.5) mean cognitive empathy score and first year students had (14.4 ± 4.8) mean cognitive empathy (Table 3 ). Year of study and mean cognitive empathy score had significant statistical association (t = −5.50, df = 226, P-value < 0.05) (Table 4 ). The median cognitive empathy score also has shown that final year students had higher cognitive empathy score than first year students (Figure 1). There was no statistically significant difference among first year students with all remaining socio-demographic characteristics. Final year male students have scored statistically lower cognitive empathy [β = −3.9, 95%CI (6.49, −1.23)] compared with final year female students. Based on the overall regression analysis, males had significantly lower [β = −2.5, 95%CI (−4.37, −0.66)] cognitive empathy score than female students. As the number of brothers and sisters the students had increased, the cognitive empathy scored decreased significantly [β = −0.4, 95%CI (−0.65, −0.20)]. Students who were using social media like facebook had significantly higher cognitive empathy score than non-users [β = 1.9, 95%CI (0.40, 3.46)]. Final year students have scored significantly higher [β = 2.7, 95%CI (1.20, 4.13)] cognitive empathy score than first year students (Table 5 ).

**Table 4:** Mean and standard deviation score of RME-E of first year and final year medical students of Jimma University; Ethiopia, 2011

**Variable**		**Mean RME-R (SD)**		**P-value**
		**First year (n_1_ = 131)**	**Final year (n_2_ = 106)**	
**Sex**	Male	14.2(4.7)	17.2(4.3)	<0.01
	Female	16.3(4.8)	21.2(4.0)	
**Ethnicity**	Oromo	13.6(4.6)	17.0(4.4)	0.31
	Amhara	14.7(4.8)	19.5(4.7)	
	Others	15.3(5.1)	18.4(3.2)	
**Grow up with**	Mother	13.6(5.2)	17.6(4.3)	0.35
	Father	12.0(2.0)	22.8(4.6)	
	Both	14.7(4.8)	17.6(4.4)	
	Other	13.7(5.3)	16.3(5.0)	
**Position in the family**	Eldest	16.1(5.0)	17.9(5.1)	0.22
	Sandwich	14.0(4.4)	17.8(4.1)	
	Youngest	14.1(5.3)	17.5(5.0)	
**Place of living**	At home with family or relatives	14.9(4.8)	18.6(4.6)	0.03
	Not at home	14.1(4.7)	16.3(4.0)	
**Working status of mother**	Working	15.2(5.3)	18.5(4.1)	<0.01
	Housewife	13.9(4.1)	16.5(4.6)	
	out of work	17.3(5.6)	20.8(4.4)	
**Working status of father**	Working	14.5(4.8)	17.6(4.3)	0.80
	Houseman	11.7(2.9)	17.6(4.9)	
	out of work	15.6(4.3)	20.8(4.0)	
**Religion**	Christian	14.4(4.8)	17.8(4.5)	0.98
	Muslim	14.6(5.0)	17.6(4.7)	
	Other	14.5(0.7)	17.0(0.0)	
**Religion active member**	Yes	14.3(4.5)	17.9(4.7)	
	No	15.2(5.2)	17.5(4.2)	
**Major life event**	Yes	14.8(5.1)	17.3(5.1)	0.66
	No	14.2(4.7)	18.1(4.1)	
**Online social media like facebook**	Yes	15.8(4.4)	17.8(4.5)	<0.001
	No	13.5(4.9)	17.6(4.3)	
**Migration history**	Yes	14.8(5.1)	17.3(5.1)	0.86
	No	14.2(4.7)	18.1(4.1)	
**Daily internet time**	less than 1 h	14.4(5.1)	17.8(4.8)	0.35
	more than 1 h	14.8(4.9)	18.2(3.9)	
	no internet	14.4(3.7)	16.8(3.6)	
**Interested in**	continuity of patient care	13.9(4.9)	18.2(4.2)	0.13
	less interpersonal contact	16.2(4.7)	18.4(4.9)	
	no idea	14.0(4.3)	16.14(4.1)	
**History of Psychiatric treatment**	Yes	13.6(7.8)	14.4(4.6)	0.11
	No	14.6(4.6)	18.1(4.4)	
**Total BEES mean (SD)**		14.4(4.8)	17.8(4.5)	<0.001

**Table 5:** Predictors of RME-E score of first year and final year medical students of Jimma University; Ethiopia, 2011

**Variables**	**β**	**Std. Error**	**95% Confidence Interval for β**	
			**Lower Bound**	**Upper Bound**
**Number of brothers and sisters**	−0.43	0.12	−0.65	−0.20
**Religion** (Reference = Muslim)				
Christian	−0.54	0.78	−2.08	1.00
Other	−0.80	2.73	−6.19	4.59
**Sex** (Reference = female)				
Male	−2.51	0.94	−4.37	−0.66
**Year of study** (Reference = first)				
Second year	2.66	0.74	1.20	4.13
**Migration history (reference = yes)**				
No	2.83	1.81	−0.75	6.40
**Grown up with (Reference = Mother)**				
Father	0.45	2.13	−3.76	4.65
Both	1.32	1.20	−1.05	3.69
Other	0.03	1.69	−3.30	3.36
**Position in the family** (Reference = Eldest)				
Sandwich	0.44	0.72	−0.98	1.85
Youngest	−1.13	0.91	−2.93	0.68
**Major life event** (reference = No)				
Yes	0.41	0.73	−1.02	1.84
**Mothers job** (reference = working)				
Housewife	−0.80	0.68	−2.14	0.53
Out of work	3.17	1.51	0.19	6.15
**Fathers job** (reference = working)				
Houseman	−0.04	1.28	−2.56	2.48
Out of work	−1.19	1.90	−4.94	2.56
Religion active member **(reference = No)**				
Yes	−0.79	0.67	−2.10	0.53
**Use of social media like facebook** (reference = No)				
Yes	1.93	0.78	0.40	3.46
**Daily internet use** (reference = no internet)				
Less than 1 h	−0.58	0.88	−2.31	1.15
more than 1 h	−0.33	1.25	−2.79	2.13
**Interested in (reference = less interpersonal contact)**				
in patient care	−0.46	0.75	−1.95	1.02
No idea	−1.68	0.99	−3.63	0.27
**History of psychiatric treatment** (reference = No)				
Yes	−2.16	1.18	−4.48	0.16
R^2^ = 0.33				

### Reliability of BEES and RME-R

The BEES and RME-R measures had acceptable reliability with Cronbach’s alpha of 0.72 and 0.70 respectively. There was weak positive correlation (Pearson correlation (r) =0.29) between emotional and cognitive empathy measures in this study.

## Discussion

Our study has found that there was no significant difference in emotional empathy between first year and final year medical students. But there was significantly higher cognitive empathy among final year students as compared to first year students. Sex was a predictor variable for both cognitive and emotional empathy. In addition, future specialization interest area and being active user of online social media like facebook were also predictor variables for emotional empathy among medical students.

Compared with the stated norms of empathy used by an instruments used for this study [32,34], there was lower mean emotional and cognitive empathy scores among Ethiopian first year and final year medical students. Preserving the low emotional empathy at final year similar to first year, may be a positive result of the students training and experience through clinical years of the students. It has been stated that ‘emotional relationships that elicit emotional response are conceptually more relevant to sympathy than to empathy’ [12].

Both cross-sectional and longitudinal studies have indicated that females have generally more emotional empathy than males [16,19,21-26]. According to psychoanalytic and evolutionary theory of parental investment, women are believed to develop greater care-giving attitudes toward their offspring than men [35] and these caring characteristics can be associated with high emotional empathy. Even the norm for empathy measure has set much higher empathy score of female than male [32]. As it has been indicated with all the above findings, male students had much lower emotional empathy scores in this particular study. But gender based comparative study is required with representative proportions to make inference in this regard.

Even though we found that final year students have scored statistically higher cognitive empathy score than first year students, practically the difference was below the standard; according to RME-R test, a mean score under 22 indicates low score [34]. This may be related with the validity of the instruments used to the culture of the study participants (the instrument used was with a Caucasian faces in the photos). In any case, final year students had higher cognitive empathy score than first year students. Similar to emotional empathy score, this may be also associated with the training in medical education or experience during the clinical years. Similar studies have found contradicting findings in this regard; studies in Japan and Korea found the highest values for measures of empathy, by year of medical school, among senior medical students [7,36] while another study in Iran did not find variations in empathy [37]. This difference might be attributed to the instruments used; since, in the Korean study they measured clinical empathy than general empathy.

Unlike that of emotional empathy, females have scored higher cognitive empathy than the males which may suggest that female may provide a better type of medical care [38-40] based on a better understanding of the patient’s experiences and feelings (cognitive empathy). In a number of studies, a higher empathy level in females was found as compared to males [4,5,7,16,19,21-26].

 Even though many findings of studies recorded a decline in empathy during medical school proceedings [3-5,16-22], in a normal circumstances we expect emotional empathy to decrease and cognitive empathy to increase as students progress through the years of medical school training. Our finding is also compatible with such a normal situation. The weak positive correlation between the two measures may be indicative of this explanation.

Previous studies found an association between the choice of medical students’ future specialization and their empathy level scores [4,22,26,27]. In our study, we found that students who did not decide about future interest specialization area had low emotional empathy than who have decided which may be attributed to first year students may not be familiar about some of the medical specialization areas and were still in undecided situation.

Students who were using online social media like facebook had significantly higher both emotional and cognitive empathy score as non-users. There was no documented previous study on the effect of using social media like facebook on empathy. Hence, further study is required to give more explanations for such differences. Another issue that needs further study is why there was inverse relationship between cognitive empathy and the number of brothers and sisters students had.

There was no statistically significant difference with other socio-demographic variables in this study. This may be due to similarity of the different cultures with regard to empathy in Ethiopia. One of the strength of this study is we have tried to measure two dimensions of empathy and the method of data collection was self administered study so that there may be less social desirability bias. Since the study is of explanatory nature, it is not worth adjusting for multiple testing. The validity of the study may be limited by a cross-sectional rather than longitudinal design of the study. The relatively small sample size and the fact that both instruments of the emotional and cognitive empathy scales were not validated in Ethiopia are the main limitations of this study. Nonetheless, this is the first study in the area and we believe it will add valuable information to the existing knowledge gap.

## Conclusions

 Low emotional BEES score and cognitive empathy score (RME-R test score) was found in first year and final year students of Jimma University may imply that the medical school curricula should improve training in empathy skills. Females were more emotional to internalize the pains and also understand the feelings of others more easily from the eyes than males. Medical education targeted at enhancing appropriate emotional empathy and increasing cognitive empathy is required by segmenting with gender of the medical students for effective physician-patient interaction. To assure all these differences on patient care, the association between empathy and clinical competence should be studied with more rigorous designs. The issue of gender-based differences in medical care given by male and female medical students needs to be investigated. Culturally validated instrument is also required for further studies of empathy.

## Competing interests

The authors declare that they have no competing interests.

## Authors’ contributions

MS was involved in the designed the study. SG was involved in the design of the study and collection of the data. SM was involved in the design of the study and analysis of the data. EG was involved in the collection, analysis of the data, drafting the manuscript and critically reviewing the manuscript. SD and MT were involved in design of the study, analysis of the data and critically reviewing the manuscript. All authors read and approved the final manuscript.

## Pre-publication history

The pre-publication history for this paper can be accessed here:

http://www.biomedcentral.com/1472-6920/12/34/prepub
